# Massed prolonged exposure for posttraumatic stress disorder among individuals in comprehensive substance use treatment: Study protocol for a randomized controlled trial

**DOI:** 10.1016/j.conctc.2026.101668

**Published:** 2026-07-08

**Authors:** Jordan A. Gette, Denise A. Hien, Shannon M. Kehle-Forbes, Steven Curto, Candace C. Hodgkins, David B. Nelson, Sonya B. Norman

**Affiliations:** a University of Kansas Medical Center, Department of Psychiatry and Behavioral Science, Kansas City, KS, USA; b Cofrin Logan Center for Addiction Research and Treatment, University of Kansas, Lawrence, KS, USA; c Center of Alcohol and Substance Use Studies, Rutgers University, New Brunswick, NJ, USA; d University of Minnesota Medical School, Minneapolis, MN, USA; e Gateway Community Services Inc, Jacksonville, FL, USA; f University of California, San Diego School of Medicine, La Jolla, CA, USA

**Keywords:** Posttraumatic stress disorder, Substance use disorders, Prolonged exposure, Massed prolonged exposure

## Abstract

Posttraumatic stress disorder (PTSD) and substance use disorders (SUDs) often co-occur and are associated with a more severe clinical profile than either disorder alone. Prolonged Exposure (PE) is empirically supported treatment for PTSD and has demonstrated effectiveness among those with a co-occurring SUD. However, PE is not typically offered in substance use treatment settings, in part due to concerns about feasibility, retention, and symptom exacerbation, as well as structural misfit with time-limited residential and outpatient programs. Massed Prolonged Exposure (M-PE), PE delivered multiple times per week and completed within a condensed timeframe, yields comparable treatment outcomes to PE and may better align with the structure of residential and outpatient care and address key barriers to implementation. Here, we describe an ongoing randomized controlled trial investigating the efficacy of M-PE versus trauma treatment as usual (TAU) delivered concurrent to SUD TAU, among individuals with co-occurring PTSD and substance use disorders enrolled in a comprehensive residential and outpatient SUD program. In addition to examining treatment outcomes, this trial utilizes qualitative methods to gain a deeper understanding of participants’ experiences and evaluate M-PE implementation barriers and facilitators in an SUD treatment setting. We describe the development and implementation of this randomized clinical trial.

## 1 Introduction

Posttraumatic stress disorder (PTSD) and substance use disorders (SUDs) frequently co-occur with more than 40% of individuals with PTSD meeting criteria for an SUD and 35% of those with an SUD meeting criteria for PTSD [[1], [2], [3]]. PTSD + SUD is associated with worse clinical outcomes than either disorder alone and these individuals have higher rates of additional comorbid psychiatric diagnoses, higher rates of suicide attempts, and greater functional impairment across social, vocational, and health domains [1,2,[4], [5], [6], [7], [8], [9], [10], [11]].

Prolonged Exposure (PE) has demonstrated effectiveness in the treatment of PTSD + SUD [12,13]. PE is a gold-standard trauma-focused intervention for PTSD that addresses trauma-related avoidance and cognitions (e.g., “I cannot trust anyone,” “the world is completely unsafe”) through use of imaginal and in-vivo exposures [14,15]. To date, there have been more than 20 clinical trials supporting the efficacy and effectiveness of PE [4]. These trials have resulted in large effect sizes with nearly three-quarters of individuals demonstrating treatment gains and 40% no longer meeting PTSD criteria by the end of treatment. Among those with PTSD + SUD, PE is more effective in reducing PTSD symptoms than SUD-only treatment and more effective than non-trauma-focused interventions for PTSD + SUD [[16], [17], [18], [19]]. Importantly, PE is well-tolerated among this population with no indication that PE results in higher rates of relapse, substance use, or symptom severity compared to treatment as usual (TAU) [12,13,20].

Despite increasing empirical support for PE among PTSD + SUD populations, PE is not typically offered in SUD treatment settings. This gap is likely driven by multiple influences including clinician beliefs regarding patients’ ability to manage exposure therapy and programmatic barriers such as poor retention and high patient dropout rates [16,21,22]. Further, PE is typically offered as 12 weekly individual sessions; however, many SUD treatment settings are time-limited (e.g., 30-, 60-, or 90-day residential and intensive outpatient programs) which may not align with a typical PE schedule. Residential and comprehensive outpatient (outpatient settings offering multiple individual/group therapy sessions per week in addition to medical, social, and/or family support) settings could serve as an opportunity to increase access to trauma-focused care as comprehensive outpatient formats are associated with higher rates of treatment attendance and completion rates [[23], [24], [25], [26]]. PTSD treatment offered through comprehensive outpatient programs is associated with lower dropout rates and longer abstinence periods [27,28]. Modifying PE to fit outpatient and residential SUD contexts could lead to enhanced treatment outcomes and greater access to care for those with PTSD + SUD.

One such modification is to offer PE in massed format. Massed PE (M-PE) offers sessions 3-5 times per week such that patients can complete treatment in 2-5 weeks as opposed to 3-5 months. There is growing support for M-PE with M-PE demonstrating higher retention, lower treatment dropout, higher session attendance, and similar treatment gains compared to weekly PE [[29], [30], [31]]. Further, offering M-PE in residential and comprehensive outpatient programs has the potential to expand access to trauma-focused care for individuals who might otherwise be unable to engage in standard outpatient PE as these programs are designed to provide frequent, structured treatment contact, often five or more days per week in residential care and up to five days per week in comprehensive outpatient care. Pilot work has shown M-PE to be feasible and acceptable in comprehensive outpatient settings with pilot work demonstrating lower dropout and greater PTSD and depression symptom reductions compared to treatment as usual (TAU) at 3-month follow-up [23].

The current study aims to expand upon this work as the first randomized hybrid Type I efficacy/effectiveness clinical trial of M-PE in a residential and comprehensive outpatient SUD treatment facility. This trial will compare M-PE + SUD TAU to trauma TAU + SUD TAU (henceforth M-PE v. TAU) among individuals with PTSD + SUD at an SUD treatment facility in the Southeastern United States. We will assess changes in PTSD symptoms, substance use outcomes (e.g., number of use days, substance-related problems), depressive symptoms, suicidal ideation, and quality of life among 168 patients enrolled in a residential or comprehensive outpatient SUD program. Further, we aim to assess potential treatment outcome moderators and mediators such as reductions in substance craving and trauma-related cognitions [[32], [33], [34], [35]]. A mixed-methods approach including qualitative interviews of participants, study therapists, and study site staff will be used to evaluate the implementation and acceptability of M-PE within the SUD program.

## 2 Design and methods

We are currently conducting a randomized (two condition) single center ([blinded for review]) controlled trial of M-PE v. TAU for individuals with PTSD enrolled in a community SUD treatment program. Following a baseline assessment confirming diagnoses of PTSD and an SUD, participants are randomized via REDCap to receive TAU or M-PE. In addition to the treatment protocol, qualitative interviews will be conducted with a purposive sample of treatment site staff and study participants. The study was approved by the Institutional Review Board of [blinded for review] and is registered at ClinicalTrials.gov (NCT06968832).

### 2.1 Participants

#### 2.1.1 Intervention participants

Projected enrollment is 168 individuals with PTSD + SUD over five years with approximately equal disruption between sex. Potential participants are identified through electronic medical records, mental health intakes, and/or referrals by providers at [site]. Participants are recruited from the residential and comprehensive outpatient programs and pre-screened for eligibility by the treatment site study coordinator. Inclusion criteria are as follows: 1) 18+ years old; 2) have full or subthreshold (missing no more than one symptom on clusters B, C, D, or E) DSM-5 PTSD; and 3) able to give informed consent. Exclusion criteria are as follows: 1) impaired mental status, 2) significant current risk of suicidal/homicidal behavior, 3) unmanaged psychotic or bipolar disorder, or 4) refusal to be audio-recorded. Given the length of study participation and the high rates of comorbid medical and mental health conditions with PTSD and SUD, individuals may receive other medical and mental health care (e.g., support groups, family therapy, psychiatric medications) while participating in the study. Individuals consent to baseline assessment and those who are eligible are consented to treatment prior to randomization.

#### 2.1.2 Qualitative interviews

Prior to study enrollment, staff members at the intervention site (*n* = 15) were invited to complete individual qualitative interviews co-produced by a Community Advisory Board to 1) capture experiences prior to study onset and 2) identify any potential gaps in the study protocol prior to implementation (e.g., therapist scheduling needs, participant transportation needs). To capture a variety of roles and expertise, all members at the treatment site were invited to participate. To maintain anonymity and avoid coercion, interested participants contacted an external research coordinator to schedule interviews and all interviews were conducted by study staff unaffiliated with the treatment site. Members of the study team from the treatment site were not provided information about qualitative interview participants. Following study completion, these individuals, along with all study therapists, will be invited to participate in a follow-up interview.

A second series of qualitative interviews will be conducted with research participants. A subset of participants randomized to a treatment condition will be randomly selected and invited to participate in a qualitative interview following the end-of-treatment interview window. We anticipate interviewing approximately 12 to 15 participants from each condition or until saturation is met (*n* = 24 – 30). Participants will be eligible regardless of treatment completion status in order to capture a wide range of experiences (e.g., reasons for terminating study participation). Interviews will be conducted by research team members not affiliated with the treatment site.

### 2.2 Treatment

Eligible participants are randomly assigned to TAU or M-PE with randomization stratified by residential or outpatient treatment using randomly permuted-blocks with equivalent allocation to each treatment group. Randomization will be computer-generated with all clinical assessors blinded to treatment condition. Individual conditions are described below. For both TAU and M-PE, potential substance use during treatment is managed on a case-by-case basis by treatment site staff and decisions are made independently of this study.

#### 2.2.1 Treatment as usual (TAU)

The treatment site offers comprehensive care including detoxification, residential and outpatient programs, and recovery housing. Typical length of stay in residential care is approximately 30 to 90 days tailored to individual need. Patients in the residential program engage in care from 7:30am to 5:00pm Monday through Friday with optional programming on weekends and evenings. Typical length of comprehensive outpatient participation is person-centered with length of participation contingent upon individual need. Individuals in the outpatient program participate in group and individual therapy up to 3 days per week depending on need. Providers at the treatment site come from multidisciplinary backgrounds including licensed social workers, Master's and doctoral level clinicians, licensed marriage and family counselors, and nurse practitioners.

**SUD TAU.** Services are tailored to the patient and include individual and group therapy, 12 steps groups, family counseling, medication management, and social support services that implement a variety of evidence-based approaches such as motivational interviewing and cognitive behavioral therapy.

**Trauma TAU.** Participants engage in a resiliency, skills-based group based on adaptations of the Seeking Safety curriculum and comprised of five 90-min standalone sessions. These sessions focus on development of coping skills and symptom management without exposure to the traumatic event.

#### 2.2.2 Massed prolonged exposure (M-PE)

M-PE follows the structure of PE and includes the following components: 1) breathing retraining, 2) imaginal exposure to the traumatic event, and 3) in-vivo exposures to trauma-related avoided situations. Outside of sessions, participants practice in vivo exposures and listen to session recordings. M-PE is delivered in 60-min sessions 3-4 times per week for 12-16 sessions as indicated by participant needs. Those randomized to M-PE participate in all TAU activities in addition to meeting with their individual M-PE counselor during free periods during typical treatment hours (7:30am to 5:00pm). M-PE is delivered by treatment site providers and research therapists including Master's level therapists and licensed social workers. M-PE providers receive twice weekly supervision by a licensed, Ph.D.-level clinical psychologist and all intervention procedures are overseen by the two study principal investigators.

### 2.3 Assessments

Participants complete a baseline interview prior to treatment randomization to establish diagnoses and symptom presentation in addition to interviews at the end-of-treatment (approximately 6 weeks post-randomization) and 1-, 3-, and 6-months post-treatment. Brief self-report assessments are administered 2-3 times per week throughout the treatment course. Participants are compensated for interviews but not for treatment sessions. All assessments are administered by experienced, blinded examiners at the Master's or doctoral-level with a minimum inter-rater reliability of .80 on PTSD and SUD clinical interviews. To determine inter-rater reliability over the study course, 10% of interviews will be randomly selected to be independently scored by licensed, Ph.D.-level researchers. Assessments include a combination of diagnostic interviews and self-report measures. The primary outcomes of interest are changes in PTSD symptoms, substance use frequency and problems, depressive symptoms, and quality of life. Additional focal assessments include measures of trauma-related cognitions and substance use craving. See Table 1 for the assessment schedule.

**Table 1:** Patient assessment schedule.

Measure	Purpose	Format	Pretreatment	During Treatment	At End of Treatment	1-, 3-, 6 Month Post-treatment
P-4 Suicide Severity Rating Scale	Eligibility, secondary outcome	Interview	X	-	X	X
Structured Clinical Interview for DSM-5	Characterize Sample	Interview	X	-	-	-
Clinician Administered PTSD Scale-5	Eligibility, Primary Outcome	Interview	X	-	X	X
Timeline Follow Back Interview	Primary Outcome	Interview	X	-	X	X
PTSD Checklist –DSM-5	Secondary Outcome, Mediator, Within Treatment Change	Self-Report	X	X	X	X
Short Inventory of Problems Revised (SIP-R)	Primary Outcome	Self-Report	X		X	X
Substance Use Inventory (SUI)	Secondary Outcome, Within Treatment Change	Self-Report	X	X		
Patient Health Questionnaire-9	Secondary Outcome	Self-Report	X	-	X	X
Brief Inventory of Psychosocial Functioning	Secondary Outcome	Self-Report	X	-	X	X
WHO – Disability Assessment Schedule	Secondary Outcome	Self-Report	X	-	X	X
Brief Substance Craving Scale	Secondary Outcome, Within Treatment Change	Self-Report	X	X	X	X
Posttraumatic Cognitions Inventory	Mediator		X	X	X	X
Treatment Services Review	Characterize Sample, Potential Covariate	Interview	-	-	X	X
Demographic Questionnaire	Characterize Sample, Moderator	Self-Report	X	-	-	-

#### 2.3.1 Assessor administered measures: baseline and follow-up interviews

**PTSD Diagnostic Interview**. The clinician-administered PTSD Scale for DSM 5 (CAPS-5) [36] is the primary PTSD symptom measure. At baseline, the CAPS-5 is used to determine presence of PTSD and pre-treatment PTSD severity. Items are rated on both frequency and intensity to derive a severity score for each symptom ranging from 0 (absent) to 4 (extreme). Items scored at a severity of 2 (moderate) or higher are considered “present” and counted toward the total number of symptoms required for each DSM-5 symptom cluster. DSM-5 requires presence of 1 or more re-experiencing symptoms (cluster B), 1 or more avoidance symptoms (cluster C), 2 or more alterations in cognition or mood symptoms (cluster D), and 2 or more arousal symptoms (cluster E). The present study allows individuals to be subthreshold by 1 symptom. The CAPS-5 also assesses dissociation symptoms, social and occupational functioning, and response validity.

**Substance Use Interviews**. The Structured Clinical Interview for DSM-5 (SCID) [37] is used to assess for presence of SUD(s) at baseline. The Timeline-Follow back (TLFB) [38] is used to assess frequency of substance use and percent of substance use days. At baseline, the TLFB assesses the 90 days prior to baseline. Subsequent interviews assess the period between the last assessment and the current interview date to allow for coverage of all days of the study period. During the TLFB, participants note any dates in which they were in a controlled environment (e.g., residential care, detoxification, hospitalization, incarceration).

**Additional Interview Measures**. Suicidal ideation is assessed using the P4 [39]. This interview assesses current suicidal ideation, plans, and intent as well as history of suicidal thoughts and behaviors. The Treatment Services Review (TSR) [40] assesses participation in various interventions (e.g., individual therapy, residential treatment, detoxification, psychopharmacological intervention) as a total count of times each service was received since the last interview.

#### 2.3.2 Self-report measures: baseline and follow-up surveys

**PTSD Measures.** The PTSD Checklist for DSM-5 (PCL-5) [41,42] is a 20-item self-report measure capturing DSM-5 PTSD Criterion B-E. Items are rated on a 1 to 5 scale with higher scores indicating greater PTSD symptom severity. The Posttraumatic Cognitions Inventory (PTCI) [43] is a 39-item self-report measure capturing cognitions related to traumatic event exposure. Items are rated on a 1 to 7 scale with higher values indicating greater negative cognitions.

**Substance Use Measures.** The Brief Craving Scale (BCS) [44] is a 16-item measure assessing current craving intensity and frequency. The Short Inventory of Problems Revised (SIP-R) [45] is a 17-item measure assessing substance use frequency and associated problems. Items are rated on a 1 to 4 scale with higher values indicating more substance-related problems. The Substance Use Inventory (SUI) [46] is a 7-item measure capturing the quantity and frequency of alcohol and substance since last assessment.

**Treatment Satisfaction.** At the end-of-treatment assessment, participants complete the Client Satisfaction Questionnaire (CSQ-8) [47]. This 8-item self-report measure assesses participant satisfaction with services provided. Items are rated on a 1 to 4 scale with greater values indicating greater satisfaction.

**Additional Measures.** Demographic characteristics are captured at baseline. Depressive symptoms are assessed using the 9-item Patient Health Questionnaire (PHQ-9) [48]. This measure captures depressive symptoms on a 0 to 3 scale with higher scores indicating greater depressive symptoms. The Brief Inventory of Psychosocial Functioning (BIPF) [49,50] is used to assess quality of life and functional impairment. This measure contains seven items rated on a 1 to 7 scale with higher values indicating greater psychosocial impairment. The WHO Disability Schedule 2.0 (WHODAS) [51] is used to assess functional impairment. This 12-item measure assesses impairment in cognition, mobility, self-care, getting along, life activities, and daily life participation.

#### 2.3.3 Self-report measures: in-treatment surveys

In-treatment self-report assessments include the PCL-5, PTCI, BCS, and SUI. This assessment takes approximately 10-15 min to complete. For those randomized to M-PE, in-treatment assessments occur prior to each M-PE session. For participants randomized to TAU, in-treatment assessments are administered twice weekly over 6 weeks to reflect the M-PE survey schedule.

#### 2.3.4 Reliability assessment

Assessment interviews are audio-recorded and saved to a secure, HIPAA-compliant cloud-based server. Over the course of the study, 10% of interviews will be randomly selected and scored by an independent evaluator. Interrater reliability will be at .80 or higher. If reliability falls below .80, the supervisor will conduct joint interviews with the assessors until a level of .80 or higher is reached in three consecutive interviews.

### 2.4 Qualitative interviews

Qualitative interviews were developed by the study team with feedback from the [blinded for review] community advisory board. All qualitative interviews are audio-recorded and transcribed with participant consent using Zoom. Interview recordings and transcriptions are saved to a secure, Health Insurance Portability and Accountability Act (HIPAA) compliant cloud-based server. Participants are compensated following completion of the interview.

#### 2.4.1 Study site interviews

Two semi-structured interview guides were developed to capture staff experiences with trauma-informed treatments and perspectives on offering concurrent trauma and substance use treatment. The pre-trial interview guide spans 11 domains including familiarity with PE, perceived benefits and risks of PE among those in SUD treatment, feasibility of incorporating trauma interventions into SUD care, and barriers and facilitators of these interventions within their treatment center. The post-trial interview guide will assess domains relating to implementation of PE including barriers, facilitators, and staff perspectives on the utility of M-PE.

#### 2.4.2 Post-treatment participant interviews

Two semi-structured interview guides were developed for the participant interviews with items tailored to the treatment condition. Interviews span several domains including perspectives on treatment offerings, barriers to receiving care, perceived benefits and shortcomings of treatment, and reasons for engaging or withdrawing from treatment.

### 2.5 Design considerations

PE is typically delivered in 90-min sessions. We elected to deliver M-PE in 60-min sessions for several reasons 1) recent studies show PE to be as effective with 60 min sessions [52,53], 2) we have found shorter PE sessions to be effective in our own SUD/PTSD treatment research which condenses PE to 60-75 min to make room for SUD intervention [54,55], 3) Berenz et al. used 60-min sessions in their pilot of PE in residential SUD treatment with excellent results [25], and 4) it will be more easily implemented and sustained at [study site] and more easily disseminated to other intensive SUD treatment programs in the future using 60-min sessions. Participants are eligible to participate if they meet full or subthreshold PTSD (i.e., missing no more than one symptom). This decision was based on research demonstrating that those with subthreshold PTSD exhibit similar levels of PTSD severity and impairment to those meeting full criteria [[56], [57], [58], [59]]. Additionally, we considered comparing M-PE to other existing trauma-focused therapies (e.g., Cognitive Processing Therapy). However, comparing M-PE to TAU allows for assessment of how the addition of evidence-based trauma therapy may enhance existing treatment programs.

### 2.6 Therapist training

Therapists complete a 2-3-day PE training and are then assigned a training case to complete a full M-PE intervention prior to seeing research participants. For training cases, therapists record all M-PE sessions. Recordings are then listened to, rated for fidelity, and reviewed in twice weekly consultation with a licensed doctoral-level clinical psychologist. The consultant meets regularly with the study MPIs to provide feedback and determine additional training needs prior to assigning therapists to see research participants. Throughout the trial, all M-PE sessions are recorded and twice weekly consultation is ongoing with additional therapist training as needed.

### 2.7 Fidelity

Fidelity assessment is two-fold: 1) therapist adherence to the intervention and 2) therapist competency in the delivery of the intervention. Adherence and competency are monitored by clinical consultation with a licensed clinical psychologist, including review of recorded M-PE sessions in addition to evaluation by an independent evaluator. The following standards are used to assess fidelity: 1) treatment manuals with weekly objectives, outcomes, and agendas, 2) therapist training, and 3) ongoing evaluation of treatment fidelity through audio-rating of therapy sessions and supervision. Twice weekly consultation is used to provide corrective feedback as needed and the supervising psychologist completes fidelity rating sheets for each session for the first case post-training. An independent evaluator will rate 10% of sessions at random. Throughout the study course, study therapists keep a session-by-session tracking form with self-ratings regarding use of the manual and covering relevant content. Adverse events (AEs) are monitored at every study contact. Potential AEs are entered into REDCap for review by the two PIs and reported as indicated by institutional and NIH policies.

### 2.8 Data analysis

#### 2.8.1 Data management

Self-report data is captured directly into Qualtrics via anonymous participant links. Interview data is entered into Qualtrics by a member of the study team at regular intervals. Data entry and quality assurance are ongoing with validation and double-entry procedures in place to ensure accurate entry. Additionally, all available data are extracted twice per year and uploaded to the National Data Archives (NDA) following the procedures outlined by the NDA.

#### 2.8.2 Treatment outcomes analyses

Primary outcomes comprise the CAPS-5 PTSD severity and TLFB percent days of substance use. PTSD-related cognitions and substance use craving will be examined as potential outcome mediators of intervention effects on the primary outcomes, the remaining follow-up measures discussed above comprise secondary outcomes. Generalized linear mixed models (GLMMs) will be used to model treatment outcomes with the form of GLMM chosen to well-model the respective outcome. The models will include fixed effects for follow-up time, treatment condition, and their interaction together with random intercepts for participants. We expect outcomes to vary by gender, age, race/ethnicity, and baseline PTSD severity so the models will include these as additional fixed effects. Missing data will be addressed using multiple imputation by chained regressions within intervention arms. Wald tests will assess whether there is an interaction between treatment condition and time of follow-up using results aggregated over models fit to the imputed datasets; if there is no such interaction, a Wald test will assess whether there is a main effect for treatment condition.

Secondary exploratory analyses will assess gender as a potential moderator of treatment effects as work has suggested women may show greater treatment gains in trauma-focused therapies compared to men and women have higher rates of developing PTSD prior to an SUD compared to men [27,60]. The potential mediating roles of changes in PTSD-related cognitions and substance use craving on treatment outcomes will be investigated and we will investigate whether changes in PTSD symptomology mediate subsequent changes in substance use outcomes. We will implement multiple analyses to investigate these potential mediation pathways. For each follow-up, we will fit generalized linear models (GLM) for the mediators and outcomes to implement the potential outcomes causal mediation analyses described by Imai et al. [61] These analyses will generate estimates of average direct and average causal mediation effects of the intervention on the outcomes. Alternate longitudinal causal mediation analyses, particularly those examining time-lagged effects of changes in PTSD symptomatology on subsequent SUD outcomes, will develop and employ marginal structural models.

Sample size was determined using Monte Carlo simulation considering uniform 30% dropout over time, correlations between outcomes across time of .30, a group standardized effect for PTSD symptoms and days substance use of .58 (Cohen's d) based on our previous research, and a .05 significance level. A sample size of 168 subjects provides >80% power to identify between group effects under these conditions.

#### 2.8.3 Qualitative analyses

Qualitative interviews will be analyzed using a rapid qualitative analysis framework [62,63]. The approach includes: 1) summarizing each interview using a template that will include separate areas for each main topic of inquiry and unexpected findings, 2) creating a data matrix that organizes the summary points for each main topic, 3) holding an analytic meeting for investigators and the interviewing team to provide their impression of the matrix contents, and 4) creating a final analytic memo summarizing the findings and noting key themes. Those summarizing interviews will conduct and discuss several summaries together to ensure consistency. Once consistency is achieved, remaining interviews will be coded by one study member with regular meetings to discuss emerging themes and questions.

## 3 Discussion

PE has been demonstrated to be safe and effective among individuals with PTSD + SUD [[16], [17], [18]]. However, despite high rates of co-occurring PTSD + SUD [1,2] and evidence that PE is more effective for treating PTSD among those with PTSD + SUD than SUD treatment alone [12], PE is rarely available in substance-use treatment settings. Potential barriers to inclusion of PE are low attendance and high dropout rates [16,64,65]. One way to increase attendance is to use a massed format wherein PE is offered 3 to 5 times per week as opposed to once weekly sessions (M-PE). Early support for M-PE has shown comparable treatment outcomes and lower dropout rates compared to weekly PE [23]. Further, M-PE aligns with a typical length of stay for individuals in residential or comprehensive outpatient SUD programs. This hybrid Type I randomized controlled trial aims to investigate whether the inclusion of M-PE enhances treatment outcomes among those in SUD care compared to trauma TAU and best practices from enhancing treatment implementation.

Strengths of the study include use of a mixed methods design to capture clinical outcomes and qualitative experiences of both providers and participants. This design allows for comparison of M-PE to TAU on key PTSD and SUD outcomes while also capturing varied experiences of study site staff and participants to enhance implementation and sustainability of incorporation of trauma-focused care in SUD programming. Additionally, this study aims to examine mediators and moderators of treatment outcomes to further tailor PTSD + SUD intervention. Lastly, inclusion of individuals with subthreshold PTSD and the limited inclusion/exclusion criteria increases the generalizability of findings.

Despite these notable strengths, this study does have limitations. M-PE is designed to include 3-5 sessions per week. Though this structure works well in residential and comprehensive outpatient settings, it may be less feasible for those in outpatient SUD settings. Further, the limited inclusion/exclusion criteria is meant to enhance generalizability but also introduces potential confounds associated with co-morbid diagnoses (e.g., depression, anxiety, medical comorbidities) common in PTSD + SUD populations [66,67]. We also allow participants to engage in ongoing treatments for physical and mental health conditions which may confound treatment outcomes. Lastly, we anticipate we may see difficulties with attendance and treatment continuation as participants transition from residential to outpatient care. To address this, we have included frequent reminder calls from study staff regarding appointments, flexible treatment scheduling and assessment date ranges, and options for study-funded transportation (e.g., bus vouchers, ride shares).

There is strong empirical support for including trauma-focused interventions, such as PE, into SUD care [1,2,12]. M-PE has shown increased treatment attendance, decreased dropout, and comparable treatment outcomes to weekly PE [23,[29], [30], [31]]. This massed format may better fit the structure of residential and comprehensive outpatient SUD programming as the massed format allows for treatment completion within 3-5 weeks. This study will provide evidence as to whether the inclusion of M-PE to TAU in an SUD program enhances treatment and quality of life outcomes. Further, qualitative interviews will provide valuable information for the implementation and feasibility of M-PE in SUD care from the perspective of participants, providers, and administrators.

## CRediT authorship contribution statement

**Jordan A. Gette:** Conceptualization, Investigation, Methodology, Supervision, Visualization, Writing – original draft. **Denise A. Hien:** Conceptualization, Funding acquisition, Methodology, Project administration, Resources, Supervision, Writing – review & editing. **Shannon M. Kehle-Forbes:** Conceptualization, Funding acquisition, Investigation, Methodology, Project administration, Supervision, Writing – review & editing. **Steven Curto:** Conceptualization, Project administration, Software, Supervision, Writing – review & editing. **Candace C. Hodgkins:** Conceptualization, Funding acquisition, Project administration, Supervision, Writing – review & editing. **David B. Nelson:** Conceptualization, Data curation, Funding acquisition, Investigation, Methodology, Project administration, Software, Writing – review & editing. **Sonya B. Norman:** Conceptualization, Funding acquisition, Investigation, Methodology, Project administration, Supervision, Writing – review & editing.

## Declaration of competing interest

This work is funded by a National Institutes of Mental Health grant (R01MH132720-01A1; MPIs Hien and Norman). The authors have no additional disclosures to report.

## Data Availability

No data was used for the research described in the article.

## References

[1] Pietrzak R.H., Goldstein R.B., Southwick S.M., Grant B.F. (2011). Prevalence and Axis I comorbidity of full and partial posttraumatic stress disorder in the United States: results from wave 2 of the national epidemiologic survey on alcohol and related conditions. J. Anxiety Disord..

[2] McCarthy E., Petrakis I. (2010). Epidemiology and management of alcohol dependence in individuals with post-traumatic stress disorder. CNS Drugs.

[3] Gielen N., RemcoC Havermans, Tekelenburg M., Jansen A. (2012). Prevalence of post-traumatic stress disorder among patients with substance use disorder: it is higher than clinicians think it is. Eur. J. Psychotraumatol..

[4] O'Neil M., McDonagh M., Hsu F. (2019).

[5] McLean C.P., Foa E.B. (2013). Dissemination and implementation of prolonged exposure therapy for posttraumatic stress disorder. J. Anxiety Disord..

[6] Edens E.L., Kasprow W., Tsai J., Rosenheck R.A. (2011). Association of substance use and VA service-connected disability benefits with risk of homelessness among veterans. Am. J. Addict..

[7] Norman S.B., Haller M., Hamblen J.L., Southwick S.M., Pietrzak R.H. (2018). The burden of co-occurring alcohol use disorder and PTSD in U.S. military veterans: comorbidities, functioning, and suicidality. Psychol. Addict. Behav..

[8] Driessen M., Schulte S., Luedecke C. (2008). Trauma and PTSD in patients with alcohol, drug, or dual dependence: a multi-center study. Alcohol Clin. Exp. Res..

[9] Tate S.R., Norman S.B., McQuaid J.R., Brown S.A. (2007). Health problems of substance-dependent veterans with and those without trauma history. J. Subst. Abuse Treat..

[10] McDevitt-Murphy M.E., Williams J.L., Bracken K.L., Fields J.A., Monahan C.J., Murphy J.G. (2010). PTSD symptoms, hazardous drinking, and health functioning among U.S.OEF and OIF veterans presenting to primary care. J. Trauma Stress.

[11] Drapkin M.L., Yusko D., Yasinski C., Oslin D., Hembree E.A., Foa E.B. (2011). Baseline functioning among individuals with posttraumatic stress disorder and alcohol dependence. J. Subst. Abuse Treat..

[12] Hien D, Morgan-Lopez A, Saavedra L, et al. Project harmony: a meta-analysis with individual patient data on behavioral and pharmacologic trials for comorbid posttraumatic stress and alcohol or other drug use disorders. Am. J. Psychiatry. 180(2):155-166. doi:10.1176/appi.ajp.22010071. PMC10016363 36475373

[13] Hien D.A., Papini S., Saavedra L.M. (2024). Project harmony: a systematic review and network meta-analysis of psychotherapy and pharmacologic trials for comorbid posttraumatic stress, alcohol, and other drug use disorders. Psychol. Bull..

[14] Hamblen J.L., Norman S.B., Sonis J.H. (2019). A guide to guidelines for the treatment of posttraumatic stress disorder in adults: an update. Psychotherapy.

[15] Zalta A.K., Gillihan S.J., Fisher A.J. (2014). Change in negative cognitions associated with PTSD predicts symptom reduction in prolonged exposure. J. Consult. Clin. Psychol..

[16] Roberts N.P., Roberts P.A., Jones N., Bisson J.I. (2015). Psychological interventions for post-traumatic stress disorder and comorbid substance use disorder: a systematic review and meta-analysis. Clin. Psychol. Rev..

[17] Roberts N.P., Lotzin A., Schäfer I. (2022). A systematic review and meta-analysis of psychological interventions for comorbid post-traumatic stress disorder and substance use disorder. Eur. J. Psychotraumatol..

[18] Simpson T.L., Goldberg S.B., Louden D.K.N. (2021). Efficacy and acceptability of interventions for co-occurring PTSD and SUD: a meta-analysis. J. Anxiety Disord..

[19] Norman S.B., Trim R., Haller M. (2019). Efficacy of integrated exposure therapy vs integrated coping skills therapy for comorbid posttraumatic stress disorder and alcohol use disorder: a randomized clinical trial. JAMA Psychiatry.

[20] Hien D.A., Smith K.Z., Owens M., López-Castro T., Ruglass L.M., Papini S. (2018). Lagged effects of substance use on PTSD severity in a randomized controlled trial with modified prolonged exposure and relapse prevention. J. Consult. Clin. Psychol..

[21] Becker C.B., Zayfert C., Anderson E. (2004). A survey of psychologists' attitudes towards and utilization of exposure therapy for PTSD. Behav. Res. Ther..

[22] Pitman R.K., Altman B., Greenwald E. (1991). Psychiatric complications during flooding therapy for posttraumatic stress disorder. J. Clin. Psychiatry.

[23] Norman S.B., Davis B.C., Colvonen P.J. (2016). Prolonged exposure with veterans in a residential substance use treatment program. Cognit. Behav. Pract..

[24] McCarty D, Braude L, Lyman R, et al. Substance abuse intensive outpatient programs: assessing the evidence. Psychiatr. Serv. 65(6):718-726. doi:10.1176/appi.ps.201300249. PMC4152944 24445620

[25] Berenz E.C., Rowe L., Schumacher J.A., Stasiewicz P.R., Coffey S.F. (2012). Prolonged exposure therapy for PTSD among individuals in a residential substance use treatment program: a case series. Prof. Psychol. Res. Pract..

[26] Watkins L.E., Patton S.C., Drexler K., Rauch S.A.M., Rothbaum B.O. (2023). Clinical effectiveness of an intensive outpatient program for integrated treatment of comorbid substance abuse and mental health disorders. Cognit. Behav. Pract..

[27] Tull M.T., Gratz K.L., Coffey S.F., Weiss N.H., McDermott M.J. (2013). Examining the interactive effect of posttraumatic stress disorder, distress tolerance, and gender on residential substance use disorder treatment retention. Psychol. Addict. Behav..

[28] Manhapra A., Stefanovics E., Rosenheck R. (2015). Treatment outcomes for veterans with PTSD and substance use: impact of specific substances and achievement of abstinence. Drug Alcohol Depend..

[29] Foa E.B., McLean C.P., Zang Y. (2018). Effect of prolonged exposure therapy delivered over 2 weeks vs 8 weeks vs present-centered therapy on PTSD symptom severity in military personnel: a randomized clinical trial. JAMA.

[30] Dell L., Sbisa A.M., Forbes A. (2023). Effect of massed v. standard prolonged exposure therapy on PTSD in military personnel and veterans: a non-inferiority randomised controlled trial. Psychol. Med..

[31] Van Woudenberg C., Voorendonk E.M., Bongaerts H. (2018). Effectiveness of an intensive treatment programme combining prolonged exposure and eye movement desensitization and reprocessing for severe post-traumatic stress disorder. Eur. J. Psychotraumatol..

[32] Diehle J., Schmitt K., Daams J.G., Boer F., Lindauer R.J.L. (2014). Effects of psychotherapy on trauma-related cognitions in posttraumatic stress disorder: a meta-analysis. J. Trauma Stress.

[33] Lyons R., Haller M., Curry I., Norman S.B. (2020). The relationship between negative trauma-related cognitions and psychosocial functioning in veterans with posttraumatic stress disorder and alcohol use disorder. Subst. Abuse.

[34] Lyons R., Haller M., Rivera G., Norman S. (2020). Negative affect mediates the association between posttraumatic cognitions and craving in veterans with posttraumatic stress disorder and alcohol use disorder. J. Dual Diagn..

[35] Sliedrecht W., de Waart R., Witkiewitz K., Roozen H.G. (2019). Alcohol use disorder relapse factors: a systematic review. Psychiatry Res..

[36] Weathers F.W., Bovin M.J., Lee D.J. (2018). The clinician-administered PTSD scale for DSM–5 (CAPS-5): development and initial psychometric evaluation in military veterans. Psychol. Assess..

[37] First M.B., Williams J.B.W., Karg R.S., Spitzer R.L. (2015).

[38] Sobell L.C., Sobell M.B., Litten R.Z., Allen J.P. (1992). Measuring Alcohol Consumption.

[39] Dube P., Kurt K., Bair M.J., Theobald D., Williams L.S. (2010). The P4 screener: evaluation of a brief measure for assessing potential suicide risk in 2 randomized effectiveness trials of primary care and oncology patients. Prim. Care Companion J. Clin. Psychiatry.

[40] McLellan T., Alterman A., Cacciola J., Metzger D., O'Brien C. (1992). A new measure of substance abuse treatment... : journal of nervous & mental disease. J. Nerv. Ment. Dis..

[41] Darnell B.C., Vannini M.B.N., Morgan-López A. (2026). Psychometric evaluation of the weekly version of the PTSD checklist for DSM-5. Assessment.

[42] Bovin M.J., Marx B.P., Weathers F.W. (2016). Psychometric properties of the PTSD checklist for diagnostic and statistical manual of mental disorders–fifth edition (PCL-5) in veterans. Psychol. Assess..

[43] Foa E.B., Ehlers A., Clark D.M., Tolin D.F., Orsillo S.M. (1999). The Posttraumatic Cognitions Inventory (PTCI): development and validation. Psychol. Assess..

[44] Mezinskis J.P., Honos-Webb L., Kropp F., Somoza E. (2001). The measurement of craving. J. Addict. Dis..

[45] Kiluk B.D., Dreifuss J.A., Weiss R.D., Morgenstern J., Carroll K.M. (2013). The short inventory of problems – revised (SIP-R): psychometric properties within a large, diverse sample of substance use disorder treatment seekers. Psychol. Addict. Behav..

[46] Weiss R., Hufford C., Najavits L., Shaw S. (1995).

[47] Kelly P.J., Kyngdon F., Ingram I., Deane F.P., Baker A.L., Osborne B.A. (2018). The client satisfaction Questionnaire-8: psychometric properties in a cross-sectional survey of people attending residential substance abuse treatment. Drug Alcohol Rev..

[48] Kroenke K., Spitzer R.L., Williams J.B.W. (2001). The PHQ-9. J. Gen. Intern. Med..

[49] Kleiman S.E., Bovin M.J., Black S.K. (2020). Psychometric properties of a brief measure of posttraumatic stress disorder–related impairment: the brief inventory of psychosocial functioning. Psychol. Serv..

[50] Marx B.P., Schnurr P.P., Lunney C.A., Weathers F.W., Bovin M.J., Keane T.M. (2019).

[51] Andrews G., Kemp A., Sunderland M., Korff M.V., Ustun T.B. (2009). Normative data for the 12 item WHO disability assessment schedule 2.0. PLoS One.

[52] Nacasch N., Huppert J.D., Su Y.J. (2015). Are 60-Minute prolonged exposure sessions with 20-Minute imaginal exposure to traumatic memories sufficient to successfully treat PTSD? A randomized noninferiority clinical trial. Behav. Ther..

[53] van Minnen A., Foa E.B. (2006). The effect of imaginal exposure length on outcome of treatment for PTSD. J. Trauma Stress.

[54] Norman S.B., Trim R., Haller M. (2019). Efficacy of integrated exposure therapy vs integrated coping skills therapy for comorbid posttraumatic stress disorder and alcohol use disorder: a randomized clinical trial. JAMA Psychiatry.

[55] Ruglass L.M., Lopez-Castro T., Papini S., Killeen T., Back S.E., Hien D.A. (2017). Concurrent treatment with prolonged exposure for Co-Occurring full or subthreshold posttraumatic stress disorder and substance use disorders: a randomized clinical trial. Psychother. Psychosom..

[56] Carty J., O'Donnell M.L., Creamer M. (2006). Delayed-onset PTSD: a prospective study of injury survivors. J. Affect. Disord..

[57] El-Gabalawy R., Blaney C., Tsai J., Sumner J.A., Pietrzak R.H. (2018). Physical health conditions associated with full and subthreshold PTSD in U.S. military veterans: results from the national health and resilience in veterans study. J. Affect. Disord..

[58] Yarvis J.S., Schiess L. (2008). Subthreshold posttraumatic stress disorder (PTSD) as a predictor of depression, alcohol use, and health problems in veterans. J. Workplace Behav. Health.

[59] Zlotnick C., Franklin C.L., Zimmerman M. (2002). Does “subthreshold” posttraumatic stress disorder have any clinical relevance?. Compr. Psychiatry.

[60] Wade D., Varker T., Kartal D., Hetrick S., O'Donnell M., Forbes D. (2016). Gender difference in outcomes following trauma-focused interventions for posttraumatic stress disorder: systematic review and meta-analysis. Psychol. Trauma, Theory Res. Pract. Pol..

[61] Imai K., Keele L., Tingley D. (2010). A general approach to causal mediation analysis. Psychol. Methods.

[62] Hamilton A.B., Finley E.P. (2019). Qualitative methods in implementation research: an introduction. Psychiatry Res..

[63] Hamilton A.B. (2013). Rapid qualitative analysis: updates/developments. https://www.hsrd.research.va.gov/for_researchers/cyber_seminars/archives/video_archive.cfm?SessionID=3846.

[64] Straus E., Worley M.J., Lyons R. (2022). Examining attendance patterns across integrated therapies for posttraumatic stress disorder and alcohol use disorder. J. Anxiety Disord..

[65] Watts B.V., Schnurr P.P., Mayo L., Young-Xu Y., Weeks W.B., Friedman M.J. (2013). Meta-analysis of the efficacy of treatments for posttraumatic stress disorder. J. Clin. Psychiatry.

[66] Grant B.F., Stinson F.S., Dawson D.A. (2004). Prevalence and Co-occurrence of substance use disorders and independent mood and anxiety disorders: results from the national epidemiologic survey on alcohol and related conditions. Arch. Gen. Psychiatry.

[67] Prior K., Mills K., Ross J., Teesson M. (2017). Substance use disorders comorbid with mood and anxiety disorders in the Australian general population. Drug Alcohol Rev..

